# Antimicrobial potential of bioactive resin composites in caries management: a systematic review of *in vitro* studies

**DOI:** 10.3389/froh.2025.1625977

**Published:** 2025-10-28

**Authors:** Sara Lopes, Pedro C. Lopes, Rita Fidalgo-Pereira, Javier Flores-Fraile, Nélio Veiga, Ana T. P. C. Gomes

**Affiliations:** 1 Faculty of Dental Medicine, Universidade Católica Portuguesa, Viseu, Portugal; 2 Centre for Interdisciplinary Research in Health (CIIS), Faculty of Dental Medicine, Universidade Católica Portuguesa, Viseu, Portugal; 3 Facultad de Medicina, Departamento de Cirugía, Universidad de Salamanca, Salamanca, Spain

**Keywords:** secondary caries, resin composites, bioactive resin composites, surface roughness, microorganism adhesion

## Abstract

**Introduction:**

Secondary caries is the leading cause of failure in resin composite restorations due to biofilm accumulation. Bioactive resin composites (BRCs) release ions that promote remineralization and inhibit bacterial growth. This review compares microbial adhesion and antimicrobial effectiveness between BRCs and conventional resin composites.

**Methods:**

A systematic search was conducted in databases PubMed, Scopus, and Cochrane Library to identify *in vitro* studies evaluating bacterial adhesion and antimicrobial effect of commercially available bioactive resin composites and their comparison with conventional resin composites. Studies reporting on microbial adhesion and/or antimicrobial effects were included.

**Results:**

A total of 272 potentially relevant articles were identified. Following PRISMA guidelines, eight articles met the inclusion criteria. The studies focused on five commercially available BRCs: Activa Bioactive Restorative (ACT), Beautifil II (BE), Cention N (CN), Equia Forte (EF), and SDR Flow Plus. Most studies assessed the adhesion of *Streptococcus mutans* in isolation. While microbial adhesion was observed on both bioactive and conventional resin composites, cell viability differed, with BRCs demonstrating superior antimicrobial effects.

**Conclusion:**

Bacterial adhesion to dental restorative materials is influenced by surface roughness, hydrophilicity, chemical composition, and ion release. This review suggests that BRCs and conventional resin composites exhibit similar surface characteristics, resulting in comparable bacterial adhesion. However, BRCs show greater efficacy in reducing bacterial viability, probably due to ion release, which modulates the local microenvironment and microbial dynamics. Further research is needed to explore the broader impact of ion release on the oral microbiome and its potential role in dysbiosis and disease progression.

**Systematic Review Registration:**

OSF Registries, https://doi.org/10.17605/OSF.IO/HRKFV

## 1 Introduction

Dental caries is the most prevalent infectious disease worldwide and a major chronic condition influenced by microbial activity (1, 2). The primary cause is pH imbalance, driven by acid production from bacterial fermentation of dietary carbohydrates, leading to tooth demineralization (3). Repeated acid exposure selects for acidogenic and acid-tolerant bacteria, disrupting pH homeostasis and promoting mineral loss (4).

The etiology of dental caries involves biochemical modifications in oral biofilms and shifts in microbial composition. Aciduric species like *Streptococcus mutans* (*S. mutans*) contribute directly to caries development (5), while alkali-producing species such as *Streptococcus salivarius, Streptococcus mitis* (*S. mitis*) and *Streptococcus gordonii* (*S. gordonii*) help maintain pH balance. Additionally, some *Streptococci* produce hydrogen peroxide, inhibiting *S. mutans* growth (6, 7). Another important microorganism involved in early childhood caries and root caries is *Candida albicans* (*C. albicans*). *C. albicans* interacts synergistically with *S. mutans* in dental biofilms, where bacterial glucosyltransferases mediate fungal adhesion and enhance exopolysaccharide production, leading to increased acidogenicity, biofilm resilience, and exacerbated enamel demineralization (8). The specific interaction between *Candida* and *Streptococci* seems to be relevant to the onset and progression of caries lesions and conditions the oral microbiome in ways which are only now beginning to be understood (8). This highlights the complexity of microbial interactions in caries progression, emphasizing the importance of pH homeostasis rather than solely focusing on acid-producing bacteria in caries management (6).

The treatment involves the removal of infected tissues to prevent further progression of the disease, and the resulting defect must then be restored using various restorative materials (9).

Historically, dental amalgam was the material of choice for restoring carious teeth due to its durability, ease of manipulation, and low cost. Composed primarily of mercury combined with a powdered alloy of silver, tin, and copper, amalgam demonstrated excellent mechanical properties and longevity, particularly in posterior teeth subjected to high occlusal forces (10). However, concerns about its aesthetic limitations, environmental impact, and health risks associated with mercury exposure have led to its gradual decline in favour of alternative materials (11). In contemporary dental practice, resin composites are routinely used for direct restorations (12). These materials are favoured for their aesthetic qualities, conservative preparation requirements, and improved handling characteristics (13).

Resin composites consist of silanated inorganic fillers dispersed into an organic matrix (14). The organic matrix typically included dimethacrylate monomers, namely bisphenol A-glycidyl methacrylate (Bis-GMA), urethane dimethacrylate (UDMA), ethoxylated bisphenol A glycol dimethacrylate (Bis-EMA) or Triethylene glycol dimethacrylate (TEGDMA) (15–17). It also contains a photoinitiator system, often a combination of camphorquinone and a tertiary amine (18, 19). The inorganic filler component comprises silanized inorganic particles of different size and shapes, including spherical irregular filler particles, with materials like silica, barium glass, ytterbium fluoride or zirconia (16, 20, 21). Despite their widespread application, methacrylate-based resin composites are prone to secondary caries, which can compromise the long-term success of direct restorations (22, 23).

Sixty percent of the restorative procedures are related to the replacement of failed restorations (24).

Secondary caries are influenced by multiple factors, including the technique sensitivity of the adhesive procedure (25), the adaptation resin composite to the cavity, polymerization shrinkage (26), and occlusal stresses generated during mastication leading to mechanical degradation (27, 28). Additional contributors include surface roughness and plaque accumulation, unreacted monomers due to incomplete polymerization and the absence of antibacterial properties in resin composites (28, 29).

The accumulation of biofilm on the restoration surface and adhesive interface, which contributes to the occurrence of caries at the tooth-resin composite interface, is a frequent challenge (25). The hydrophobic nature and surface roughness resin composites create an environment conducive to biofilm formation. Cariogenic bacteria, such as *S. mutans* and *Lactobacillus spp.*, adhere to these surfaces and metabolize carbohydrates into acids, leading to localized demineralization and restoration failure (30).

In addition, the complex enzymatic composition and bacterial flora of saliva exacerbate the challenges on the oral environment (31, 32). These technical and biological challenges justify the growing interest in innovative bioactive resin composites (BRCs) for direct restorative treatments. Unlike traditional resin composites, BRCs actively interact with the oral environment to promote remineralization and reduce bacterial colonization (33). BRCs, besides the usual components of the resin composites, are also composed by calcium phosphate or fluoride-releasing fillers, that under acidic conditions are released to protect against demineralization and inhibit caries progression (21). Recent advancements, such as the integration of nanotechnology and antibacterial agents, have further improved their mechanical properties and resistance to biofilm formation (34). The incorporation of fiber reinforcement and “smart” bioactive features has expanded the scope of resin composite applications. Fiber-reinforced resin composites improve structural integrity by preventing crack propagation, especially in large posterior restorations (35). Meanwhile, smart resin composites are engineered to respond to environmental changes, releasing therapeutic ions when pH levels drop below critical thresholds, providing a dynamic defence against caries (36).

These developments highlight a paradigm shift in restorative dentistry, emphasizing materials that not only restore functionality but also actively promote oral health. This systematic review aims to critically evaluate the current literature on BRCs, with a particular focus on bacterial adhesion and antimicrobial efficacy. The objective is to provide an in-depth perspective on the advancements and challenges associated with these innovative materials in restorative dentistry. Specifically, the main objectives of this review are to compare the adhesion of cariogenic and carioprotective microorganisms to the resin composites under study and assess the effectiveness of bioactive resins in inhibiting microbial growth.

## 2 Materials and methods

This systematic review was conducted following the Preferred Reporting Items for Systematic reviews and Meta-Analysis (PRISMA) (37) guidelines and has been registered in the OSF Registries, under the registration doi: 10.17605/OSF.IO/HRKFV.

The focused question was determined using the Population, Intervention, Comparison and Outcome (PICO) strategy, formulated as “In *in vitro* studies, do commercial bioactive resins differ from conventional commercial resins in terms of microorganism adhesion and antimicrobial activity, when exposed to microorganisms?” where:

P (Participants): Commercial bioactive resin composites;

I (Intervention): Exposure to microorganisms;

C (Comparison): Commercial conventional resin composites;

O (Outcome): Adhesion of microorganisms on the surface and the antimicrobial effect of bioactive resin composites;

S (Study type): *In vitro* studies.

An electronic search was conducted in PubMed, Scopus, and Cochrane Library databases in October 2024, covering the last 10 years. A combination of keywords, including resin composites, surface properties, bacterial adhesion and biofilm, were used in the databases following their syntax rules. All combinations using (AND, OR) were utilized to refine the search results. The search key: ((composite resins [MeSH Terms) AND (surface properties [MeSH Terms) AND ((bacterial adhesion [MeSH Terms) OR [biofilm (MeSH Terms)]).

The aim is to identify articles that examine the adhesion of microorganisms to commercially available resin composites and BRCs. The articles retrieved from the three databases were exported to Rayyan - Intelligent Systematic Review (38), where the selection of articles was performed by two independent authors SL and PL. The results of the different bases were combined to eliminate duplicated documents and articles were screened by title and abstract. When the title or abstract did not provide sufficient information regarding the inclusion criteria, the full text was obtained and analyzed.

The eligibility criteria for inclusion in this review were as follows: articles published within the last 10 years; in English or Portuguese language; focused on commercially available BRCs and comparison with traditional resin composites; reports on microorganism adhesion or antibacterial effects; included findings on microorganism adhesion to the surface of the material; papers that compared the adhesion and/or antimicrobial effects of BRCs with conventional resins and studies conducted *in vitro*. The eligibility criteria for exclusion were as follows: resin composites are not commercially available; studies that involved different resin composites surface treatments (e.g., varying polishing methods, adhesive systems); combination of resin composites with adhesive systems; articles that do not address improvements in antimicrobial effects and/or adhesion of bioactive resins compared to conventional resins and articles without full-text access.

Articles that did not meet all the inclusion criteria were excluded. Any disagreement regarding the inclusion of specific articles was resolved through discussion with a third author (ATPCG). To evaluate the methodological quality of the studies, the Quality Assessment Tool for *In Vitro* Studies (QUIN Tool) was used (39). The same reviewers (SL and PL) collected the data independently, in tables structured in Excel, (Microsoft Corporation^TM^, USA) spreadsheets with essential information such as: Author, Study design, bioactive resin, resin composite (control), microorganisms in study, objectives, results and conclusion.

## 3 Results

**Figure 1:**
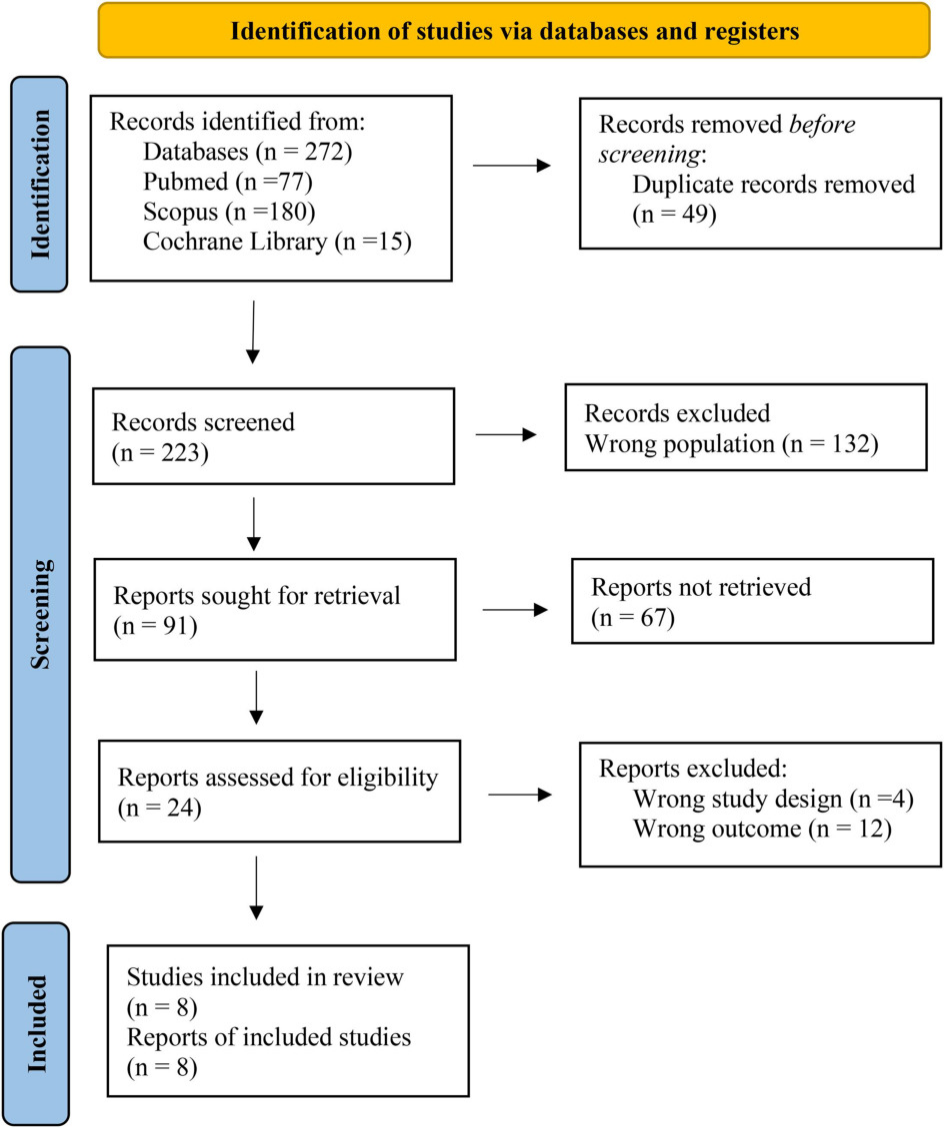
Flow PRISMA diagram of the search strategy used in the present systematic review.

The initial search yielded a total of 272 potentially relevant articles, with 77 publications from PubMed, 180 from Scopus, and 15 from Cochrane Library, of which 49 duplicate articles were eliminated. Of the remaining, 223 articles, title and abstract were read, and articles were selected according to the inclusion and exclusion criteria. Of the remaining 24 articles, full text was read, and 8 articles were considered in the current review (Figure 1).

The primary reason for excluding 132 articles was the use of experimental BRCs, whose suitability for application in the oral cavity remains unknown. Therefore, only studies involving commercially available BRCs were included.

Furthermore, 39 articles were excluded due to unsuitable study designs, and 28 were omitted since the bacterial adhesion was not evaluated, which was a key focus of this review.

This review exclusively considered *in vitro* studies in which resin composites were produced using molds without the application of adhesive techniques. Microbial adhesion was assessed on BRCs without prior surface treatment, as current literature demonstrates that polishing does not affect bacterial adhesion (40–42).

The studies selected were analyzed regarding the quality of the study according to the QUIN Tool (39). criteria and the results of the analysis are presented in Table 1.

**Table 1:** Risk of bias analysis using the QUIN tool for selected studies.

Study/Author(s)	Clearly stated aims/objectives	Detailed explanation of sample size calculation	Detailed explanation of sampling technique	Details of comparison group	Detailed explanation of methodology	Operator details	Randomization	Method of measurement of outcome	Outcome assessor details	Blinding	Statistical analysis	Presentation of results	Score	Bias evaluation
Beldüz et al. (47)	2	2	2	2	2	0	2	2	0	0	2	2	18	**75.00%** **Low risk**
Yoshihara et al. (43)	2	2	2	2	2	0	2	2	0	0	2	2	18	**75.00%** **Low risk**
Bilgili et al. (48)	2	2	2	2	2	0	2	2	0	0	2	2	18	**75.00%** **Low risk**
Daabash et al. (41)	2	2	2	2	2	0	2	2	0	0	2	2	18	**75.00%** **Low risk**
Sengupta et al. (45)	2	2	2	2	2	0	2	2	0	0	2	2	18	**75.00%** **Low risk**
Lehrkinder et al. (49)	2	2	2	2	2	0	2	2	0	0	2	2	18	**75.00%** **Low risk**
Chen et al. (46)	2	2	2	2	2	0	2	2	0	0	2	2	18	**75.00%** **Low risk**
Dey et al. (44)	2	2	2	2	2	0	2	2	0	0	2	2	18	**75.00%** **Low risk**

Adequately specified = 2; inadequately specified = 1; not specified = 0; >70% Low risk; 50%–70% = medium risk; <50% high risk of bias. The bold values indicate the bias evaluation for each domain assessed using the QUIN tool. All bold values correspond to “Low risk,” highlighting that the included studies were generally assessed as having a low risk of bias.

Among the studies included in the review, the majority focused on the adhesion of isolated microorganisms. Most of these studies assessed the adhesion of cariogenic *S. mutans* (41, 43–46) and one study in *C. albicans* (47). Only two studies evaluated multiple species: one study evaluated the adhesion of *S. mutans* and *S. mitis* in isolated forms (48), while another assessed the adhesion of multispecies cariogenic biofilm composed by *S. mutans*, *S. mitis*, *Streptococcus Salivarius* (*S. salivarius*), *Streptococcus sanguinis* (*S. Sanguinis*), and *Lactobacillus acidophilus* (*L. acidophilus*) (49)*.*

The BRCs investigated across the studies were limited to five commercially available materials: Activa Bioactive Restorative (ACT), Beautifil II (BE), Cention N (CN), Equia Forte (EF), and SDR Flow Plus. Their compositions are presented in Table 2.

**Table 2:** Composition of BRCs based on the manufacturer's specifications.

Material	Type	Resin Matrix	Filler	Manufacturer
Activa bioactive restorative (ACT)	Enhanced resin-modified glass ionomer (RMGIC)	Patented ionic resin matrix, shock-absorbing rubberized resin (diurethane and other methacrylates with modified polyacrylic acid 44.6%)	Reactive ionomer glass fillers 55.4 wt% of bioactive glass and sodium fluoride	Pulpdent, Massachusetts, US
Beautifil II (BE)	Giomer	Bis-GMA UDMA Bis-MPEPP TEGDMA	S-PRG filler based on fluoroboroaluminosilicate glass and nanofiller(10–20 nm)	Shofu, Kyoto, Japan
Cention N (CN)	Alkasite Resin Composite	UDMA, DCP, Aromatic aliphatic-UDMA, PEG-400 DMA	Barium aluminum silicate glass, Ytterbium trifluoride, Isofiller, Calcium barium aluminum fluorosilicate glass, Calcium fluorosilicate glass (78.4 wt%, 57.6 v% of inorganic filler) Particle size range of 0.1–35 μm Powder/liquid ratio (g/g) = 4.6/1.0	Ivoclar Vivadent Schaan, Liechtenstein
Equia forte (EF)	Glass Hybrid Bulk fill Restorative	Powder: Fluoro-alumino-silicate glass, polyacrylic acid, pigment. Liquid: Water, polyacrylic acid, carboxylic acid.	__________________	GC Corporation, Tokyo, Japan
SDR flow plus	Bulk fill flowable	Modified UDMA; TEGDMA; polymerizable dimethacrylate resin and polymerizable trimethacrylate resin	70.5 wt%/47.4 vol% silanated barium-alumino-fluoro-borosilicate glass; silanated strontium alumino-fluoro-silicate glass and surface treated fume silicas	Dentsply Sirona

Bis-GMA (bisphenol A-glycidyl methacrylate); Bis-MPEPP (Bisphenol A polyethoxy dimethacrylate) UDMA (urethane dimethacrylate); TEGDMA (triethyleneglycol dimethacrylate).

The conventional resin composites used for comparison by studies are: Admira FusionX-tra, Ceram X, Herculite XRV Ultra, Grandio SO, G-aenial Universal Injectable, Dyract Flowable, Filtek Z350XT, Filtek Bulk Fill, Tetric® N-Ceram. Their compositions are presented in Table 3.

**Table 3:** Composition of conventional resin composites based on the manufacturer's specifications.

Material	Type	Resin Matriz	Filler	Manufacturer
Admira FusionX-tra (AFX)	Nano-Hybrid and ORMOCER (ORganically MOdified CERamic)	BisGMA, TEGDMA or HEMA	Organically modified silicic acid, fumes silica, 2,6-di-tert-butyl-p-cresol	Voco
Ceram X (CE)	Universal nano-ceramic restorative	Methacrylate modified polysiloxane, dimethacrylate resin	Barium alumino fluoro borosilicate glass (BAFG) and nano-sized silicon dioxide particles (0.85–0.9 μm, 77% wt)	Dentsply Sirona
Dyract flowable	Compomer restorative	Phosphoric acid modified polymerizable monomers, carboxylic acid modified macromonomers	Strontium-alumino-fluoro-silicate glass	Dentsply Sirona
Herculite XRV Ultra	Universal nanohybrid	Bis-GMA, TEGDMA, BisEMA	SiO_2_, Barium silicate glass, Prepolymerized filler with barium silicate glass and silica	Kerr Corporation,
Grandio SO	Universal nano-hybrid	Bis-GMA, Bis-EMA, and TEGDMA	Glass-ceramic fillers, and silicon dioxide nanoparticles	Voco
G-aenial universal injectable	Universal	Bis-MEPP	Silicon dioxide, Barium glass	GC Corporation
Filtek Z350XT (Z350)	Nanohybrid composite	Bis-GMA, Bis-EMA, UDMA	Non aggregated 20 nm, Silica filler, nonaggregated 4–11 nm, zirconia filler, and aggregated silica/zirconia cluster filler	3M ESPE
Filtek bulk fill (FBF)	Nano composite	Bis-MEPP, Bis-GMA, and TEGDMA	Silane Treated Silica, Silane Treated Zirconia, YbF3	3 M ESPE
Sonic fill 2 (KSF)	Universal	Bis-GMA, TEGDMA, Bis-EMA	SiO2, glass, oxide	Kerr
Tetric® N-Ceram (TNC)	Nanohybrid resin composite	Bis-GMA, Bis-EMA and urethane dimethacrylate monomer (UDMA), involving advanced composite-filler technology, patented light initiator Ivocerin	Barium aluminum silicate glass with two different mean particle sizes, filler content approximately 61%(volume) and 17% polymer fillers or “Isofiller”	

Bis-GMA (bisphenol A-glycidyl methacrylate); Bis-MPEPP (Bisphenol A polyethoxy dimethacrylate) UDMA (urethane dimethacrylate); TEGDMA (triethyleneglycol dimethacrylate).

Table 4 provides an overview of the 8 studies included in this review, their objectives, the resin composites investigated, and their findings related to microbiological adhesion to the resin composites and cell viability.

**Table 4:** Summary of results from the eight studies included in this systematic review.

Author year	Aims	MO in study	BRCs	Control	Adhesion/bacterial growth	Cellular viability
Beldüz et al. 2016 (47)	To compare the susceptibility of *C. albicans* adhesion and cellular viability on restorative materials.	*Candida albicans* clinical strain (SC5314)	Beautifil II (BE)	Grandio SO	No diferences.	Low cellular vitality of BE.
Yoshihara et al. 2017 (43)	To compare the bacterial growth and ion release properties of a bioactive resin with those of a conventional resin.	*S. mutans* (ATCC25175)	Beautifil II (BE)	Herculite XRV Ultra	No diferences.	No reference.
Bilgili et al. 2020 (48)	To evaluate the *S. mutans* and *S. mitis* adhesion and related surface properties of bulk-fill resin composite.	*S. mutans* ATCC 25175 *S. mitis*	Beautifil Bulk (BE)	Sonic Fill 2 (KSF) Filtek Bulk Fill (FBF) Admira FusionX-tra (AFX)	No difference in adhesion to the materials overall. However, greater adhesion of *S. mitis* was observed, with adhesion being higher in the KSF group.	A higher number of dead bacteria were observed on the surfaces of FBF and BE.
Daabash et al. 2023 (41)	To evaluate the adhesion of *S. mutans* and related surface properties of ion-releasing resin-based composite.	*S. mutans* ATCC 25175	Cention N (CN) Activa Bioactive Restorative (ACT)	Filtek Z350 XT (Z350)	Greater adherence of S. mutans was observed on ACT.	The percentage of dead cells is higher in CN, with no significant differences observed between ACT and Z350.
Sengupta et al. 2023 (45)	To evaluate and compare bacterial adhesion properties using *S. mutans*.	*S. mutans* ATCC 25175	SDR Flow plus	Ceram X (CE)	No diferences.	No reference.
Lehrkinder et al. 2024 (49)	To analyze the impact of composite dental materials on cariogenic biofilm formation.	Biofilm samples *S. mutans* IB, *S. mitis* ATCC 4956, *S. salivarius* CCUG 17825, *S. sanguinis* CCUG 10556, *L.acidophilus* CCUG 5917	Beautifil II (BE)	CeramX (CE) Admira Fusion (AD)	Bacterial growth was observed on all materials in both pH conditions, with biofilm growth being superior at neutral pH. *S. mutans* exhibited the greatest growth compared to other species, with higher growth at acidic pH in CE. The growth of *S. salivarius* was superior on all materials at neutral pH. *S. mitis* showed the least growth at both pH levels. *S. sanguinis* exhibited greater growth at acidic pH on both BE and CE.	No reference.
Chen et al. 2024 (46)	To evaluate biocompatibility and antibacterial behaviours of injectable composite resins.	*S. mutans* ATCC 35,668™	Beautifil Injectable XSL (BE)	G-aenial Universal Injectable (GU) Filtek Supreme (FS) Dyract Flowable (DF)	Cells on GU and FS specimens exhibited favorable adhesion and active proliferation.	FS exhibited a higher bacterial density and more viable bacterial colonies, while BE showed more inactive colonies. This difference was more noticeable in FS specimens, with bacterial colonies being less dense in BE specimens.
Dey et al. 2024 (44)	To evaluate and compare bacterial adhesion on two bioactive restorative materials.	*S. mutans* ATCC 25175	Equia Forte TM HT Fil (EF) Cention N® (CN)	Tetric® N-Ceram (TNC)	A lower amount of *S. mutans* was observed in CN. In EF, the biofilm was smaller, and the *S. mutans* chains were less tightly packed and dense.	No reference.

Beldüz et al. 2016 (47), revealed a fine biofilm layer of *C. albicans* on the surfaces of all examined materials, observed through scanning electron microscopy (SEM). Metabolic activity assays further indicated that the *C. albicans* formed significantly less viable biofilms, suggesting that the properties of Beautifil II could influence microbial viability.

Building on this, Yoshihara et al. 2017 (43) evaluated the antibacterial properties and surface stability of different dental restorative materials, focusing on their ability to inhibit bacterial adhesion and biofilm formation. This work showed that Beautifil II, albeit releases ions with potential antibacterial properties, its surface degradation in acidic conditions promotes bacterial adhesion and biofilm formation. Therefore, this BRCs does not effectively inhibit bacterial growth and may even enhance biofilm formation due to increased surface roughness. Also, the conventional resin composite Herculite XRV Ultra demonstrated good surface stability but did not exhibit antibacterial properties or inhibit biofilm formation. This suggests that while it maintains its structural integrity, it does not provide additional benefits in preventing bacterial adhesion or growth. Further exploring material properties, Bilgili et al. 2020 (48) demonstrated that surface roughness did not significantly affect bacterial adhesion for both BRCs Beautifil II and the conventional Filtek Bulk Fill. However, authors reported higher surface free energy values that were associated with increased bacterial adhesion, particularly for the carioprotective *S. mitis*. Although BRCs Beautifil II did not significantly reduce bacterial adhesion compared to conventional resin composites, a greater number of dead *S. mutans* were observed on the Beautifil II surface, suggesting a potential material-related effect on bacterial viability, despite the lack of significant differences in CFU counts.

Similarly, Daabash et al. 2023 (41) examined the surface roughness and bacterial adhesion of several ion-releasing and conventional resin composites. The results showed that BRCs Cention N exhibited significantly higher surface roughness compared to Filtek Z350XT and Activa Bioactive Restorative resin composites. Despite its smooth surface, Activa Bioactive Restorative revealed the lowest antibacterial effect, as evidenced by a higher accumulation of *S. mutans* bacteria than the conventional resin composite Filtek Z350XT and the BRCs Cention N. This BRCs had a rougher surface compared to Activa Bioactive Restorative but was more effective in reducing viable bacteria.

In a similar study, Sengupta et al. 2023 (45) showed that nano-ceramic restorative material Ceram X had a smoother surface compared to the SDR Flow Plus. However, this did not translate into a significant difference in bacterial adhesion. In fact, no difference between *S. mutans* adhesion amongst these materials was observed. This suggests that other factors than surface roughness, such as surface energy, hydrophobicity, and material composition, may play a more critical role in bacterial adhesion.

Lehrkinder et al. 2024 (49) investigated the impact of different dental restorative materials on the formation of cariogenic biofilm composed of *S. mutans*, *L. acidophilus*, *S. mitis*, *S. sanguinis*, and *S. salivarius* by exposing resin composites to pH 7 (neutral) and pH 5.5 (acidic) conditions. The results showed that bacterial adhesion to dental materials were mainly influenced by surface roughness and composition rather than fluoride release. Biofilm accumulation was similar across materials, but bacterial counts varied, especially at acidic pH. Despite high fluoride release, bacterial growth was not significantly inhibited. Beautifil II is smooth surface and ion release contributed to lower bacterial adherence, suggesting its potential to reduce secondary caries risk.

Chen et al. 2024 (46) evaluated the mechanical properties, wear resistance, antibacterial behavior, and biocompatibility of several injectable composite materials. The study compares two injectable nanocomposite resins G-aenial Universal Injectable and Beautifil II, one flowable composite resin Filtek Supreme, and one flowable compomer Dyract Flowable. The results showed that injectable nanocomposites showed superior mechanical properties, wear resistance, and biocompatibility in MC3T3-E1 cells compared to the flowable compomer. Water storage negatively affected all materials. Biocompatibility tests showed reduced MC3T3-E1 cell viability, with Dyract Flowable performing worse. Antibacterial properties tests against *S. mutans* were similar across materials, though Filtek Supreme had slightly higher biofilm density.

Finally, Dey et al. 2024 (44) evaluated bacterial adhesion of *S. mutans* and surface roughness of two BRCs, Equia Forte and Cention N (an alkasite), compared to a conventional resin composite, Tetric® N-Ceram. Cention N showed the lowest bacterial adhesion, while the conventional composite had the smoothest surface. No correlation was found between surface roughness and bacterial adhesion. These findings suggest that factors like ion release play a key role, with Cention N and Equia Forte demonstrating bioactive properties that help inhibit bacterial adhesion.

Overall, the results revealed that the differences in bacterial adhesion between BRCs and conventional resin composites were not statistically significant. However, Beldüz et al. 2016 (47), Bilgili et al. 2020 (48), Daabash et al. 2023 (41) and Chen et al. 2024 (46)*,* have shown that BRCs affect bacterial cell viability, suggesting that the released ions influence bacterial growth.

## 4 Discussion

The interaction between dental restorative materials and bacterial adhesion is influenced by multiple factors, including surface roughness, chemical composition, ion release properties, and the antimicrobial potential of the resins (50). The *in vitro* studies included in this systematic review provide a comprehensive perspective on the microbial adhesion patterns and antimicrobial properties of BRCs and conventional resin composites, highlighting key findings related to their effectiveness in preventing bacterial colonization and biofilm formation. In the present review, the term bioactive resin composite refers primarily to materials containing fillers capable of ion release, such as calcium, phosphate, fluoride, or bioactive glass particles, as summarized in Table 2. The polymer matrix itself is not bioactive, but functions to incorporate and stabilize these fillers. It should be noted, however, that silanization of filler particles, which is necessary to achieve durable bonding with the resin matrix, may reduce their ion-releasing capacity.

It is well known that bacteria are more prone to adhere to hydrophilic surfaces with high surface energy, which significantly impacts the performance of resins composite (51).

Most studies reviewed indicate that BRCs do not consistently exhibit significant antimicrobial properties or reduced bacterial adhesion compared to conventional resin composites. Several investigations, including those by Beldüz et al. 2016 (47), Yoshihara et al. 2017 (43), Bilgili et al. 2020 (48), and Sengupta et al. 2023 (45), found no significant differences in bacterial adhesion between BRCs and conventional resin composites. Lehrkinder et al. 2024 (49) further demonstrated that *S. mutans* exhibited the highest growth under acidic conditions, regardless of the resin type, emphasizing that microbial colonization is heavily influenced by environmental factors.

However, some studies identified variations in bacterial adhesion depending on the specific composition of BRCs. For example, Daabash et al. 2023 (41) found increased *S. mutans* adhesion on Activa Bioactive Restorative, while Chen et al. 2024 (46) observed lower bacterial viability on Beautifil II compared to Filtek Supreme.

These findings suggest that not all bioactive materials possess inherent antimicrobial properties, and their effectiveness may depend on specific chemical compositions and environmental conditions: materials capable of releasing ions such as fluoride, calcium, or zinc can interfere with bacterial metabolism and biofilm growth, but their long-term efficacy depends on maintaining stable ion release under oral challenges, including acidic pH and surface degradation.

### 4.1 Effect of surface roughness on bacterial adhesion

Surface roughness has traditionally been considered a crucial factor in bacterial adhesion, as rougher surfaces provide more retention sites for microbial colonization (52). However, the results from several studies challenge this assumption. While Daabash et al. 2023 (41) reported that the BRCs Cention N had a rougher surface yet exhibited reduced bacterial adhesion, Sengupta et al. 2023 (45) showed that conventional resin composite Ceram X had a smoother surface than bulk-fill resin composites but demonstrated no significant differences in bacterial adhesion.

Other studies, such as those by Bilgili et al. 2020 (48) and Lehrkinder et al. 2024 (49), found that surface roughness alone does not determine microbial adhesion. Instead, additional factors, such as surface free energy and the chemical composition of the resin composite, likely play a more significant role in bacterial colonization.

These findings emphasize that while surface roughness can influence microbial adhesion, it is not the sole determinant of bacterial attachment to resin composites (53).

### 4.2 Chemical composition and its role in bacterial adhesion

The chemical composition of resin-based materials, particularly the presence of ion-releasing components, plays a significant role in bacterial adhesion and biofilm formation (54, 55). While some BRCs contain fluoride, calcium, and phosphate-releasing compounds that promote antimicrobial activity (56), the effectiveness of these components varies. Certain BRCs, such as Cention N and Beautifil II, have been shown to release fluoride, calcium, and phosphate ions, creating an unfavourable environment for bacterial adhesion. Studies, such as that by Dey et al. 2024, have observed that these ion-releasing materials exhibit lower *S. mutans* adhesion, likely due to their alkalizing effects and ability to promote remineralization.

In addition to ion release, some BRCs incorporate antibacterial nanoparticles or monomers, such as silver or zinc oxide, to reduce microbial colonization. However, not all BRCs contain these components, which may explain the inconsistent antimicrobial results observed across different studies. The hydrophilicity of the resin composite, influenced by the type of monomers used in its formulation, also plays a role in bacterial adhesion. Monomers such as Bis-GMA, UDMA, and TEGDMA affect the material's hydrophilicity (57) with more hydrophilic surfaces tending to attract bacterial biofilms, while hydrophobic materials may exhibit reduced microbial attachment. Additionally, the degree of cross-linking within the polymer network contributes to bacterial colonization, as more tightly cross-linked resins are less prone to degradation and microbial penetration (58).

These factors highlight the complexity of bacterial interactions with resin-based materials and the need for further research to optimize their antimicrobial properties.

### 4.3 Antimicrobial properties of bioactive resin composites

The antimicrobial activity of bioactive resin composites remains a subject of debate, as studies have reported varying results regarding their effectiveness in reducing microbial viability (59, 60). While some bioactive materials have demonstrated the ability to limit bacterial growth, others show minimal antimicrobial effects. Research by Daabash et al. 2023 (41) found that the BRCs Cention N exhibited lower bacterial viability despite having a rougher surface, suggesting that chemical composition and ion release may have a more significant impact on antimicrobial activity than surface texture alone. Similarly, studies by Bilgili et al. 2020 (48) and Chen et al. 2024 (46) reported higher numbers of dead bacterial cells on Beautifil II and Filtek Bulk Fill, supporting the idea that certain bioactive materials can promote bacterial death more effectively than conventional resin composites.

Further reinforcing this perspective, Dey et al. 2024 (44) observed that Equia Forte and Cention N demonstrated reduced *S. mutans* counts and biofilm formation, particularly due to their fluoride or calcium-releasing properties. However, the antimicrobial effects of BRCs are not consistently observed across all materials. Yoshihara et al. 2017 (43) found that despite the ion-releasing properties of Beautifil II, it did not effectively inhibit bacterial growth under acidic conditions, likely due to its increased surface roughness under these circumstances provided additional sites for bacterial retention, counteracting the expected antimicrobial effect.

These findings suggest that while some BRCs can influence bacterial viability through ion release and surface properties, their antimicrobial potential ultimately depends on the materiaĺs ability to maintain these functions over time, which is governed by their chemical stability and resistance to environmental degradation.

This systematic review presents several limitations that should be acknowledged. First, all included studies were conducted *in vitro*, which, although controlled, do not fully replicate the complex biological and mechanical conditions present in the oral cavity. This limits the direct applicability of the findings to clinical practice. Second, there was considerable heterogeneity among the studies in terms of methodology, including differences in microbial strains used, testing protocols, incubation periods, and outcome measures, which prevented meaningful quantitative comparison. In particular, the lack of standardization in how microbial adhesion and cell viability were measured across studies further complicates direct comparison and synthesis of results. Moreover, the microbial spectrum was narrow, with most studies focusing solely on *S. mutans*, while few assessed multispecies biofilms or other cariogenic microorganisms. Additionally, only five types of commercially available bioactive resin composites were investigated, which may not represent the full range of materials used in clinical practice.

Thus, although ion release is often highlighted during the commercial presentation of BRCs as a key contributor to their antimicrobial potential, the available scientific evidence remains largely indirect and inconsistent. Reported concentrations released from fillers embedded in the resin matrix appear too limited to ensure a sustained effect, and the process of silanization—while essential for mechanical reinforcement—may further reduce ion availability. These limitations suggest that ion release alone may not fully account for the antimicrobial effects observed, emphasizing the need for further well-designed studies to clarify its role.

These limitations highlight the need for standardized, long-term, and clinically relevant studies to better understand the antibacterial performance of bioactive resin composites.

## 5 Conclusion

The studies reviewed highlight that bacterial adhesion to dental restorative materials is not solely determined by surface roughness but is significantly influenced by chemical composition, ion release properties, and material hydrophilicity. While BRCs have the potential to reduce microbial viability through ion release and antimicrobial agents, their effectiveness remains inconsistent across different formulations.

To enhance the antimicrobial performance of restorative resin composites, future research should focus on systematically evaluating the effectiveness of ion release by considering both quantity and duration, while also exploring the incorporation of antibacterial agents and strategies to balance mechanical durability with surface stability. By addressing these factors, the development of more effective bioactive restorative materials may contribute to improved clinical outcomes and enhanced resistance to bacterial colonization. Nevertheless, while BRCs hold promise for reducing bacterial viability and contributing to caries prevention, further standardized, long-term *in vivo* studies are essential to validate their clinical efficacy and guide evidence-based material selection in restorative dentistry.

## Data Availability

The original contributions presented in the study are included in the article/Supplementary Material, further inquiries can be directed to the corresponding authors.
